# Day-to-day intrapersonal variability in mobility patterns and association with perceived stress: A cross-sectional study using GPS from 122 individuals in three European cities

**DOI:** 10.1016/j.ssmph.2022.101172

**Published:** 2022-07-16

**Authors:** Jonathan R. Olsen, Natalie Nicholls, Fiona Caryl, Juan Orjuela Mendoza, Luc Int Panis, Evi Dons, Michelle Laeremans, Arnout Standaert, Duncan Lee, Ione Avila-Palencia, Audrey de Nazelle, Mark Nieuwenhuijsen, Richard Mitchell

**Affiliations:** a MRC/CSO Social and Public Health Sciences, University of Glasgow, Glasgow, United Kingdom; b Transport Studies Unit, University of Oxford, Oxford, United Kingdom; c Hasselt University, Centre for Environmental Sciences (CMK), Hasselt, Belgium; d Flemish Institute for Technological Research (VITO), Mol, Belgium; e School of Mathematics and Statistics, University of Glasgow, Glasgow, United Kingdom; f Queen's University Belfast, Belfast, United Kingdom; g Centre for Environmental Policy, Imperial College London, London, United Kingdom; h MRC-PHE Centre for Environment and Health, Imperial College London, United Kingdom; i Barcelona Institute for Global Health (ISGlobal), Barcelona, Spain; j Universität Pompeu Fabra (UPF), Barcelona, Spain; k CIBER Epidemiología y Salud Pública (CIBERESP), Spain

**Keywords:** Human mobility, Day-to-day variability, Intrapersonal variability, Mental health, Perceived stress, Urban health

## Abstract

Many aspects of our life are related to our mobility patterns and individuals can exhibit strong tendencies towards routine in their daily lives. Intrapersonal day-to-day variability in mobility patterns has been associated with mental health outcomes. The study aims were: (a) calculate intrapersonal day-to-day variability in mobility metrics for three cities; (b) explore interpersonal variability in mobility metrics by sex, season and city, and (c) describe intrapersonal variability in mobility and their association with perceived stress.

Data came from the Physical Activity through Sustainable Transport Approaches (PASTA) project, 122 eligible adults wore location measurement devices over 7-consecutive days, on three occasions during 2015 (Antwerp: 41, Barcelona: 41, London: 40). Participants completed the Short Form Perceived Stress Scale (PSS-4). Day-to-day variability in mobility was explored via six mobility metrics using distance of GPS point from home (meters:m), distance travelled between consecutive GPS points (m) and energy expenditure (metabolic equivalents:METs) of each GPS point collected (n = 3,372,919). A Kruskal-Wallis H test determined whether the median daily mobility metrics differed by city, sex and season. Variance in correlation quantified day-to-day intrapersonal variability in mobility. Levene's tests or Kruskal-Wallis tests were applied to assess intrapersonal variability in mobility and perceived stress.

There were differences in daily distance travelled, maximum distance from home and METS between individuals by sex, season and, for proportion of time at home also, by city. Intrapersonal variability across all mobility metrics were highly correlated; individuals had daily routines and largely stuck to them. We did not observe any association between stress and mobility.

Individuals are habitual in their daily mobility patterns. This is useful for estimating environmental exposures and in fuelling simulation studies.

## Highlights

• Many aspects of our daily life are related to our mobility patterns and individuals can exhibit tendencies towards routine.
• Day-to-day variability in mobility was explored via six mobility metrics calculated from GPS data (n = . 3,372,919).
• Individuals were habitual in their daily mobility patterns.
• There were differences in intrapersonal mobility patterns by sex, season and by city.
• We did not observe any association between stress and mobility.

## 1 Introduction

Almost half of daily behaviours can be classed as being habitual (McCloskey & Johnson, 2021); individual habits and routines may be deliberately constructed within specific time-periods where individuals can exhibit strong tendencies towards routine in their daily lives (Ersche et al., 2017; Ramakrishnan et al., 2021). Habituality of human mobility, referred to as day-to-day or intrapersonal variability, has been well studied within the transportation literature, including description of transportation/travel patterns (Pas & Koppelman, 1986; Zhang et al., 2021), active travel patterns (Joh et al., 2002; Raux et al., 2016), physical activity levels (Aarts et al., 1997; Abel et al., 2019) and to evaluate transport interventions (Fan et al., 2020), for example. Less is known about the association of day-to-day variability in human mobility with mental health. Understanding human mobility, where people go and when (Montanari, 2005), is significant for public health to maximise the impact of health interventions (Oliver et al., 2015). If people are in the same places day-to-day it helps us understand exposure and how to target them for intervention. There is a need for future research to understand the geographies of mobility that take into account the effects of people's mobility on their health and well-being (Kwan & Schwanen, 2016).

Urban form, characterised by the density, size, shape and configuration of settlements, can influence human mobility behaviour. Individuals living in large or less compact cities travel greater daily distances (Kang et al., 2012) to the extent that travel patterns of residents can be used to predict the population densities of cities (Lee & Holme, 2015). Urban form can also influence people's energy expenditure. For example, those living in mixed use neighbourhoods are more likely to have greater levels of physical activity that may benefit health (Oliveira, 2016). Additional factors can influence mobility patterns; for example active transportation can exhibit seasonal trends, which is higher during summer months compared to winter (Yang et al., 2011). There are gender differences in mobility behaviour; females have been shown to both travel less and have a lower cycling prevalence than males (Shaw et al., 2020). We may expect both intrapersonal variation across seasons and interpersonal variation by gender. Further exploration of variability in mobility patterns within individuals and between individuals residing in different cities by season and sex is required.

Aggregated daily distance travelled and energy expenditure measures provide useful representation of mobility patterns. Home is a significant location where adults spend most of their time (Siła-Nowicka et al., 2016) and individual travel behaviours are very frequently anchored to this location. For example, if an individual makes the same journey from home to work every weekday, we expect their daily distance travelled to be similar. A second key measure is daily energy expenditure. The number, distance and mode of trips contribute to an individual's total daily energy expenditure (Chaix et al., 2014). The pattern and similarity of daily energy expenditure tells us what kind of mobility a person has and how their choices vary from day-to-day. Aggregated day-to-day variation in distances travelled has shown to be important for measuring mental health outcomes in pilot studies (Depp et al., 2019; Raugh et al., 2020) but further investigation for the energy expenditure measure is required. That is why it was included as an outcome in this study.

In contrast to group-level measures of mobility, individual-level day-to-day variability and between-individual variability in behaviours are often described as intrapersonal and interpersonal variability respectively (Dharmowijoyo et al., 2014). Intrapersonal variability describes the variability of mobility *within* an individual over specific time-frames, such as day-to-day, and interpersonal variability describes the variability *between* individuals across socioeconomic or built environment attributes (Zhang et al., 2021). Quantifying day-to-day variability in mobility patterns enables it to be explored, compared and associated with other characteristics including, for example, health (Barlacchi et al., 2017; Yang et al., 2021).

Studies have examined day-to-day variability in mobility patterns and mental health outcomes, referred to as digital phenotyping (Jacobson et al., 2019; Kleiman et al., 2018), however these are largely limited by selective sample biases or have focused on specific mental health conditions (Birk & Samuel, 2020). Studies have shown that decreased day-to-day mobility is associated with increasing depressive symptoms (Faurholt-Jepsen et al., 2014) and individuals with greater day-to-day variability in their behaviours also reported lower levels of depression and loneliness (Müller et al., 2020). It has recently been proposed that Global Positioning System (GPS) mobility information could act as a digital biomarker of negative mental health outcomes (Depp et al., 2019), suggesting that when an individual suffers from any kind of health condition this will be expressed in digital traces recorded in data (Birk & Samuel, 2020). Two studies examined day-to-day variability of aggregated GPS-derived metrics for adults with schizophrenia, both finding that people with schizophrenia travelled shorter daily distances compared to healthy comparators (Depp et al., 2019; Raugh et al., 2020). A smaller study in the United Kingdom (UK) recorded mobility data using a smart-phone based app for 28 adults and observed a significant correlation between mobility patterns and depressive mood (Canzian & Musolesi, 2015). Larger studies have shown that mobility patterns provided good predictions of mental health issues within small homogeneous student samples, but these predictions are only slighter higher than chance within a nationally representative sample (Müller et al., 2021). Suggesting further investigation between different population groups is required.

Few studies have explored both intrapersonal and interpersonal variability in mobility patterns for different geographical contexts linked to mental health outcomes, and these have focused mainly on intrapersonal day-to-day variability (Birk & Samuel, 2020; Depp et al., 2019; Faurholt-Jepsen et al., 2014; Mueller et al., 2018; Raugh et al., 2020). The specific objectives of this study were therefore to:

a. Calculate intrapersonal day-to-day variability in mobility metrics for Antwerp, Barcelona and London.
b. Describe individuals' patterns of mobility using detailed data, collected using GPS data from Antwerp, Barcelona and London.
c. Explore interpersonal variability in mobility metrics by sex, season and city.
d. Describe day-to-day intrapersonal variability in mobility and their association with mental health outcomes.

## 2 Material and methods

### 2.1 Study setting

This study obtained data from the FP7 PASTA project (Physical Activity through Sustainable Transport Approaches), which has been described previously (Dons et al., 2015, 2017). GPS data were collected for three cities: Antwerp, Barcelona and London. There is variation in the urban form of the cities in terms of size (Antwerp: 204 km^2^; Barcelona: 99 km^2^; London: 1594 km^2^) and population density (Antwerp: 2567 per km^2^; Barcelona: 37027 per km^2^; London: 5562 per km^2^) (European Commission, 2020). The proportion of land cover within each city varied for green urban areas (Antwerp: 5%; Barcelona: 6%; London: 10%); industrial, public or commercial (Antwerp: 8%; Barcelona: 19%; London: 11%); and railways or fast transit roads (Antwerp: 8%; Barcelona: 2%; London: 1%) (Copernicus, 2018). There is variation in the age dependency ratio (population aged 0–19 and 65 and more to population aged 20–64) between cities (Antwerp: 69; Barcelona: 62; London: 57), further city characteristics are provided in Supplementary Table 1.

The three cities were chosen due to diversity in built environments, travel habits of their residents and for pragmatic reasons of the leading research institutions responsible for administering and running the surveys being located in these cities, further detail is provided in the study protocol paper (Gerike et al., 2016).

### 2.2 Dataset

From the PASTA sample, 122 eligible and willing respondents were selected in three cities to wear GPS (I-GOTU GT-600) and SenseWear (model MF-SW, BodyMedia, Pittsburgh, PA) devices for location and physical activity measurement over 7 consecutive days, on three occasions during 2015 (Antwerp: 41 participants, Barcelona: 41 participants, London: 40 participants) to represent 3 seasons: winter, summer and mid-season (autumn or spring). All real-time sensor data were aggregated to a 1 min time resolution (Dons et al., 2017). The GPS data were run through the Physical Activity Location Measurement System (PALMS) algorithm, a web-accessible system enabling the development of travel behaviour and physical activity variables from GPS device data (Kang et al., 2018), for cleaning and detecting locations (Bekö et al., 2015; Tandon et al., 2013), described in more detail by OrjuelaMendoza (2018). The SenseWear (software version 8.0) armband is a multi-sensor body monitor that measures heat flux, galvanic skin response, skin temperature and 3-axis accelerometry. Age, sex, body weight and height of the participants are provided manually to the SenseWear professional software (version 8.0).

Whilst the PASTA participants are skewed towards higher socio-economic positions (Laeremans et al., 2017) and there are 40 participants per city, the international nature of the data (giving variety in urban form and infrastructure) offer great potential for exploring intrapersonal and interpersonal variability in mobility. Additional strengths were: one study design applied across all sites, a large number of GPS locations recorded (n = 3,372,919), length of wear-time achieved and accompanying extensive individual-level data capturing participants’ characteristics (such as sex, age, and income).

**Fig. 1:**
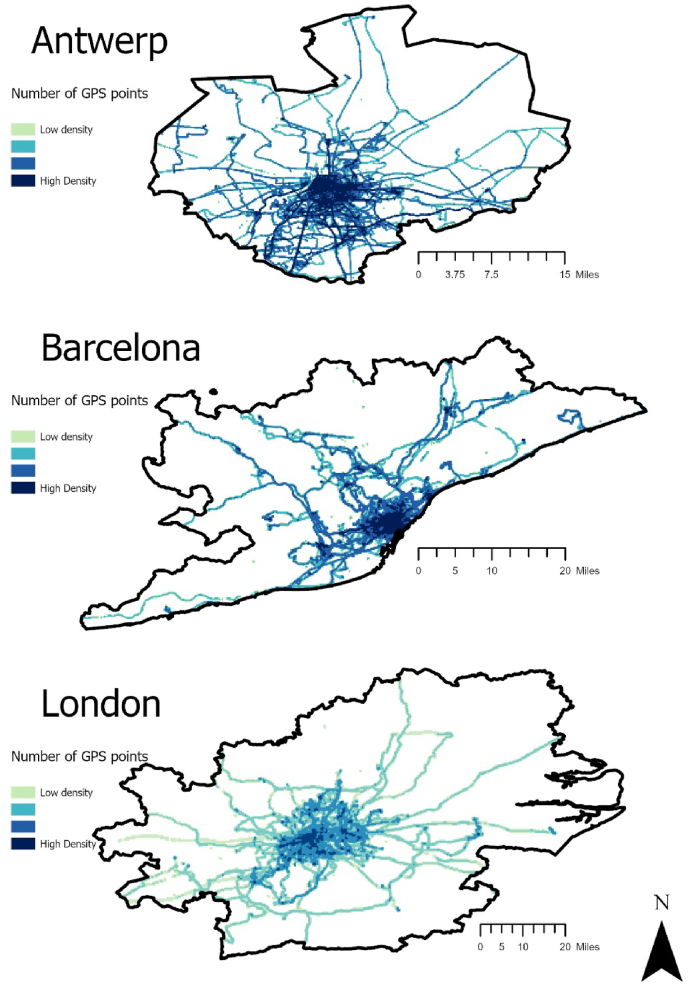
Geographical distribution and density of GPS points by city.

There was an even distribution of GPS data collection by city (Antwerp: 1,089,596 (32% of total); Barcelona: 1,151,244 (34%); London: 1,132,079 (34%)), sex (Male: 1,476,202 (56%); Female: 1,896,717 (44%)) and season (Winter: 1,074,907 (29.6%); Spring: 731,182 (20.1%); Summer: 934,247 (25.7%); Autumn: 896,061 (24.6%)). Fig. 1 presents the geographical distribution and density of GPS points by city.

### 2.3 Mobility metrics

**Fig. 2:**
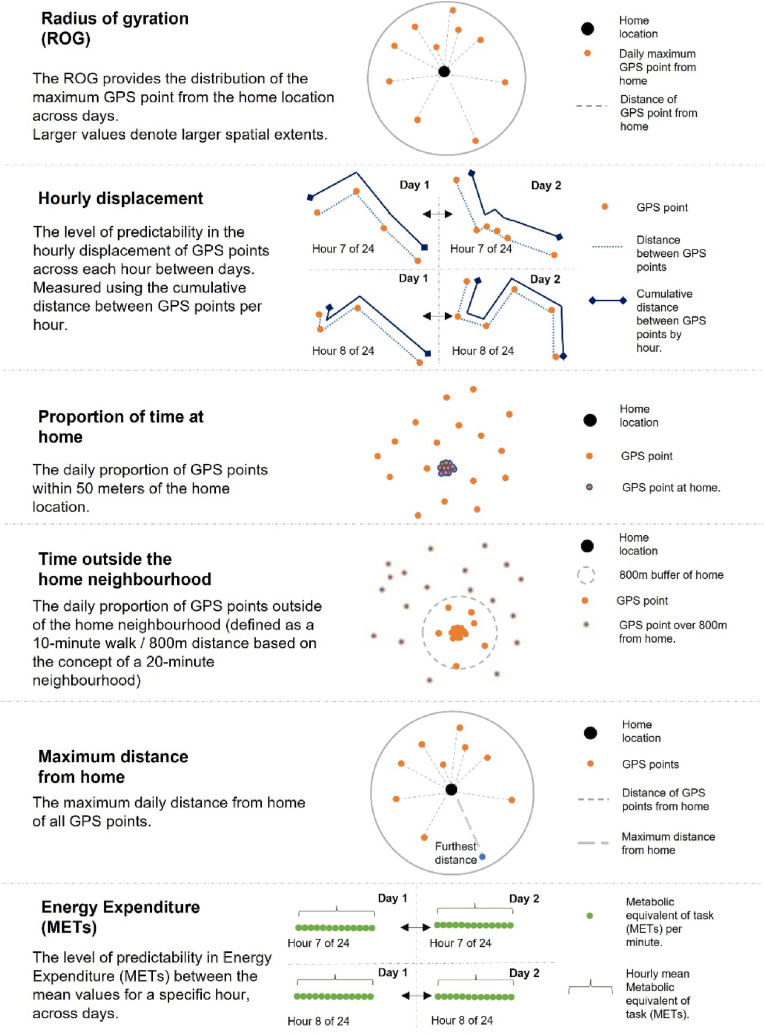
Description and visual explanation of mobility metrics.

Day-to-day variability was explored for six mobility metrics: radius of gyration (ROG); hourly displacement; percentage of time at home; time outside the home neighbourhood; maximum distance from home; and energy expenditure. The mobility metrics were selected as they are commonly applied to calculate unique elements of human mobility (Heiler et al., 2020; Long & Ren, 2022; Müller et al., 2021; Zhao et al., 2019) and are described in more detail within Fig. 2, including a visual explanation. A 50 m buffer around the home location was used to define whether a GPS point was at the home location to account for the home footprint and GPS scatter (Crist et al., 2021). A 800 m buffer surrounding the home was used to define the home neighbourhood based on a 10-min walk from home, commonly applied to represent a 20-min neighbourhood (Olsen, Nicholls, et al., 2022).

Three key mobility measures were calculated to create the mobility metrics:

*Straight line distance from home*: The straight-line distance from all GPS point locations (longitude and latitude) to each individuals’ home location in meters (m) was measured using the *geosphere* package in R version 3.5.1 (Hijmans et al., 2017). Home locations were provided by participants and geocoded.

Displacement between locations: The straight-line distances between consecutive GPS point locations were measured (m) and the total distance travelled summarised per hour and daily for each individual.

*Energy expenditure (Metabolic equivalent of task (METs))*: The SenseWear calculates METs (the ratio of the energy cost during a specific activity to the energy cost at rest) on a 1-min basis using proprietary algorithms based on pattern recognition and these were temporally linked to each GPS point (Laeremans et al., 2017).

#### 2.3.1 Mental health outcome (perceived stress scale (PSS-4))

The mental health outcome used was the four-item Short Form Perceived Stress Scale (PSS-4). PSS-4 score was collected once for each user from an online questionnaire administered during the final measurement week (Avila-Palencia et al., 2018). The PSS-4 is a global measure of stress that is simple to use and has been validated in a variety of settings and in multiple languages (Warttig et al., 2013). The PSS-4 has also been shown to be a useful instrument for assessing stress perception levels in different European countries (Vallejo et al., 2018). Scores can range from 0 to 16, with higher scores indicating higher perceived stress.

### 2.4 Statistical analysis

#### 2.4.1 Summary statistics of mobility variables

We summarised and described the six mobility variables (ROG, distance travelled (cumulative between GPS points), percentage time at home, percentage time outside the home neighbourhood, maximum distance from home and METs) daily for individuals by age, sex, season and city. Due to nonnormal distribution of daily values, medians values were used and meteorological season (Summer, Autumn, Winter and Spring) was classed from the date GPS information was recorded. A Kruskal-Wallis H test was conducted to determine if the mobility outcomes differed by city, sex and season.

#### 2.4.2 Intrapersonal and interpersonal day-to-day variability in mobility

Fig. 2 provides a visual description outlining how daily outcome measures were computed for six mobility metrics. We provide additional detail here of how we computed the ROG and measures between hours across days (energy expenditure and hourly displacement), the ROG here is given by:

$$\sqrt{\frac{\sum d^{2}}{n}}$$

where d is maximum distance from home each hour and n is the number of maximum distances recorded per day (24 h were not always recorded).

For energy expenditure and displacement within each hour (hourly displacement), first the level of predictability across each hour between days was calculated. Hour by hour mean values for METs and displacement were created for each individual, for each day. We then computed the correlation between the values for a specific hour, across days. For example, the correlation was calculated between MET values for every 9am–10 am h recorded for that individual, in that season. These coefficients captured, at an individual-level, the between-day variability in METS and displacement for each hour. Matrices were then produced for each individual to assess hour-by-hour variation in correlations across all days of measurement.

The similarity in behaviour based on proportions of time spent at home/outside and correlations in energy expenditure/displacement within an hour are described by variances, as given by:

$$\frac{\sum {\left(x-\bar{\;x}\right)}^{2}}{n-1}$$

where n is the number of days and x depends on the metric and can be correlation in average METS per hour between each day; correlation in distance travelled per hour between each day; proportion of time spent in the “at home” location; and proportion of time spent outside the home neighbourhood.

**Fig. 3:**
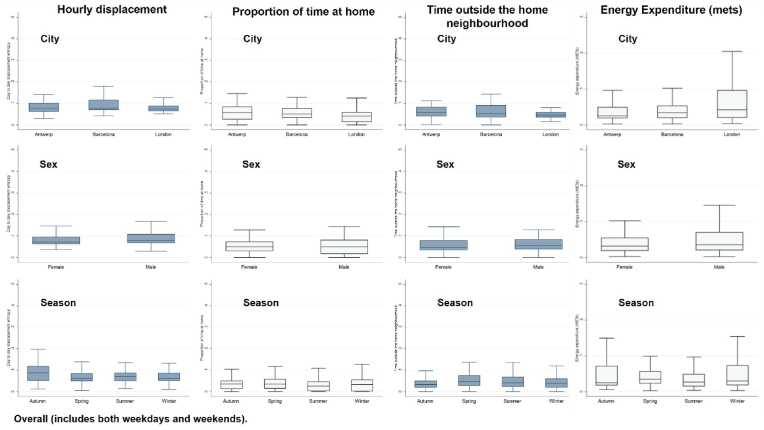
Day-to-day variance across all individuals in daily correlation coefficient by season, sex and city: hourly displacement, proportion of time at home, time outside the home neighbourhood and energy expenditure (mets), overall (includes both weekdays and weekends).

For all variance-based metrics, a small value indicated little day-to-day difference between the mobility patterns. Variance was calculated as an overall weekly value and for weekdays (Monday to Friday) only to allow for the fact that many people work weekdays and may follow a specific routine during this period (Mehrotra & Musolesi, 2017). Due to limited data collection (data for one weekend period collected) we could not compare differences between weekends separately. The variances were determined for each season separately, as well as combined. ROG and maximum distance from home are described by medians, as there are no upper bounds for these metrics and can take any positive value and zero, with all individuals on different scales. An outlier or where an individual simply travelled double the distance of another can skew the variance to be large when in fact the behavioural patterns are not irregular. Variance is appropriate within an individual but cannot be computed or summarised between individuals and therefore was not summarised in Table 3 or Fig. 3.

Intrapersonal variation in mobility was compared by sex, season and city. The day-to-day variance across all individuals for hourly displacement, proportion of time at home, time outside the home neighbourhood and energy expenditure (mets) are summarised by box plots presenting the median and interquartile range by season, sex and city.

#### 2.4.3 Intrapersonal day-to-day variability in mobility and perceived stress

The PSS-4 score does not have a clinically determined cut-off point to denote mental ill-health. In the absence of cut off points, groups were created based on tertiles of the available scores. Data were available for 94 of the 122 individuals, and tertiles categorised the scores as follows: low stress (PSS-4: 0–3), middle (PSS-4: 4 to 5) and high stress (PSS-4: 6 and over).

The intrapersonal variances in mobility were then described for each PSS-4 tertile. As the PSS-4 scores were collected at one time point (survey end), the overall intrapersonal variation score was used.

Levene's test (Levene, 1960) is one of several that can be used to ensure that the equality in group variances assumption is met for ANOVA, before comparing groups means. Given this, it was considered that it could be used to test whether the variances between the PSS4 groups were indeed different. However, Levene's test requires that the observations themselves are independent, which was not the case for our data, so for the variances of the correlations and proportions of time, the generalised Levene's Scale test was used (Soave & Sun, 2017) which allows for correlation between the observations, with participant specified as the grouping element. The Kruskal-Wallis test (Kruskal & Wallis, 1952) is suitable for comparing medians between groups, and thus was used to compare median ROG and maximum distance travelled from home between the PSS4 groups.

Analysis was run in Stata version 16.1 (StataCorp., 2019), and R version 4.0.5, using packages *lubridate*, *data.table, gJLS* and *Hmisc*. (Dowle & Srinivasan, 2021; Grolemund & Wickham, 2011; Jr, 2021; R Core Team, 2021).

## 3 Results

### 3.1 Participant characteristics and mobility patterns

A total of 122 individuals wore GPS devices for 7 consecutive days over 3 periods in one calendar year (2015–2016). The participants were from Antwerp (n = 41), Barcelona (n = 41) and London (n = 40). 54.9% (n = 67) of participants were male, the median age was 33 years, and 93.4% (n = 114) reported they were in good, very good or excellent health (Table 1). 72% (n = 88) of participants were in full-time employment, 16% (n = 20) students and half (53%, n = 65) had no children aged 17 years or under living at home. Further information of participants by city is provided in Table 1.

**Table 1:** Sample description.

	Antwerp	Barcelona	London	Total
	n (%)	n (%)	n (%)	n (%)
**Sex**				
Male	18 (43.9)	25 (61.0)	24 (60.0)	67 (54.9)
Female	23 (56.1)	16 (39.0)	16 (40.0)	55 (45.1)
**Age (years)**				
Median	36	33	31	33
Range	19 to 59	19 to 59	18 to 60	18 to 60
**Self-rated health**				
Excellent/V good/good (%)	39 (95.1)	35 (85.4)	40 (100)	114 (93.4)
**Number of children living at home**				
0 aged less than 17 years of age	17 (41.5)	20 (48.8)	28 (70.0)	65 (53.3)
1 or more aged less than 6 years of age	11 (26.8)	12 (29.3)	5 (12.5)	28 (23.0)
1 or more aged 6–17 years of age	13 (31.7)	9 (22.0)	7 (17.5)	29 (23.8)
**Employment status**				
Full-time employment	33 (80.49)	32 (78.05)	23 (57.5)	88 (72.13)
Part-time employment	4 (9.76)	2 (4.88)	3 (7.5)	9 (7.38)
Student	4 (9.76)	5 (12.2)	11 (27.5)	20 (16.39)
Unemployed/Retired/Parental leave	0 (0)	2 (4.88)	3 (7.5)	5 (4.1)
Household income (after tax (£0,000))*				
Less than 24	7 (18.4)	8 (25.8)	4 (11.5)	19 (18.3)
25 to 49	19 (50.0)	14 (45.2)	8 (22.9)	41 (39.4)
More than 50	12 (31.6)	9 (29)	22 (62.9)	43 (41.4)
Prefer not to answer	0 (0)	0 (0)	1 (2.9)	1 (1.0)
Total	41 (100)	41 (100)	40 (100)	122 (100)

Note: *Household income groups <10 and 10 to 24 combined due to small numbers in these groups. Missing household income data for Antwerp (3), Barcelona (10) and London (5).

The median daily distance travelled of all GPS points for all users was 12392.5 m (Table 2). Kruskal-Wallis H tests showed that there were differences in ROG, daily distance travelled, maximum distance from home and METS between individuals by sex, season and, for proportion of time at home also, by city. For example, among individuals living in London the median daily distance travelled was 14106.4 m, whereas it was 12956.8 m in Antwerp, and 9976.8 m in Barcelona. Daily distance travelled for males (14835.8 m) were greater than for females (10679.3 m). The median daily mean METS across cities was 1.57, the equivalent of the lower level of light physical activity (Jetté et al., 1990), and there was variation by season of measurement, with the highest energy expenditure occurring in spring (1.62) and lowest in winter and autumn (1.55).

**Table 2:** Individual daily mobility summaries by season, city and sex.

Daily summaries (number of individuals; number of daily observations)	Radius of gyration (ROG)		Distance travelled (m)		Proportion of time at home (%)		Time outside the home neighbourhood (%)		Maximum distance from home (m)		Energy expenditure (METs)	
	Median	Range	Median	Range	Median	Range	Median	Range	Median	Range	Median	Range
**Total**												
All (n = 122; obs = 2279)	3482.3	2.0 to 222096.2	12392.5	0 to 2071840	30.6	0 to 100	40.0	0 to 100	6535.4	2 to 260632	1.57	0.88 to 5.89
**City**												
Antwerp (n = 41; obs = 732)	3484.2	2.0 to 70552.3	12956.8	0 to 799132	33.9	0 to 100	38.8	0 to 100	6489.2	2 to 133649	1.63	0.88 to 3.79
Barcelona (n = 41; obs = 764)	2609.8	7.2 to 167967.1	9976.8	0 to 639167	28.4	0 to 100	39.0	0 to 100	4748.9	7 to 181975	1.51	0.93 to 4.75
London (n = 40; obs 783)	4726.6	7.2 to 222096.2	14106.4	0 to 2071840	26.2	0 to 100	41.4	0 to 100	8482.5	7 to 260632	1.60	0.92 to 5.89
*Kruskal-Wallis H test*	χ2(2) = 16.350, p = 0.0003		χ2(2) = 16.988, p = 0.0002		χ2(2) = 17.548, p = 0.0002		χ2(2) = 3.796, p = 0.1499		χ2(2) = 18.843, p = 0.0001		χ2(2) = 66.279, p = 0.0001	
**Sex**												
Male (n = 67; obs = 985)	4718.3	7.2 to 167967.1	14835.8	0 to 1614717	28.9	0 to 100	40.1	0 to 100	8541.6	7 to 181975	1.69	0.93 to 5.89
Female (n = 55; obs = 1294)	2674.3	2.0 to 222096.2	10679.3	0 to 2071840	31.1	0 to 100	39.4	0 to 100	5173.8	2 to 260632	1.50	0.88 to 4.17
*Kruskal-Wallis H test*	χ2(1) = 22.088, p = 0.0001		χ2(1) = 22.088, p = 0.0001		χ2(1) = 0.136, p = 0.7123		χ2(1) = 1.344, p = 0.2464		χ2(1) = 29.611, p = 0.0001		χ2(1) = 197.387, p = 0.0001	
**Season**												
Winter (obs = 724)	3358.3	15.2 to 222096.2	11310.1	0 to 344333.9	28.3	0 to 100	40.1	0 to 100	6359.6	15 to 260632	1.55	0.88 to 4.17
Spring (obs = 466)	3512.3	2.0 to 109703.8	11652.8	0 to 407592.1	28.6	0 to 100	41.8	0 to 100	6060.3	2 to 129271	1.62	0.93 to 4.75
Summer (obs = 593)	3385.9	7.2 to 111248.3	11140.4	0 to 222840.4	35.4	0 to 100	38.1	0 to 100	6575.4	7 to 137575	1.58	0.97 to 5.89
Autumn (obs = 496)	3917.8	15.8 to 167967.1	15759.2	0 to 2071840	27.9	0 to 100	40.7	0 to 100	7730.5	15 to 208033	1.55	0.92 to 3.79
*Kruskal-Wallis H test*	χ2(3) = 10.590, p = 0.0142		χ2(3) = 38.307, p = 0.0001		χ2(3) = 7.424, p = 0.0595		χ2(3) = 6.549, p = 0.866		χ2(3) = 11.812, p = 0.0081		χ2(3) = 18.459, p = 0.0004	

### 3.2 Day-to-day variation in mobility measures

Table 3 summarises the variance in intrapersonal day-to-day correlation coefficients for all participants, overall and for weekdays only, for four mobility metrics: hourly displacement, proportion of time at home, time outside the home neighbourhood and energy expenditure (METS). A variance of 0 indicated no variability identified across the daily correlation coefficients; a person with a variance of 0.2 has twice the amount of variation in behaviour as someone with variance of 0.1. Overall, variance in day-to-day hourly displacement (0.076) was 1.7 times higher than variance in METs (0.042) (i.e., day-to-day hourly displacement was slightly more variable than energy expenditure: Table 3). However, all figures are relatively close to zero, suggesting that the sample followed similar daily patterns of mobility, even in different weeks (seasons) of the study.

**Table 3:** Day-to-day variance across all individuals in daily correlation coefficient: day-to-day hourly displacement (m), proportion of time at home, time outside the home neighbourhood and energy expenditure (mets), overall and weekdays only.

Measure	Hourly displacement		Proportion of time at home (%)		Time outside the home neighbourhood (%)		Energy Expenditure (METs)	
	Median	Range	Median	Range	Median	Range	Median	Range
All	0.076	0.03 to 0.26	0.05	0 to 0.146	0.052	0 to 0.186	0.042	0.01 to 0.28
Weekday only	0.067	0.025 to 0.206	0.036	0 to 0.192	0.042	0 to .205	0.021	0.00 to 0.39

Note: 0 variance indicates no variability identified across individuals' daily correlation coefficients, the closer to zero, the more regular the daily mobility. Variances are absolute to compare, a person with a variance of 0.2 has twice the amount of variability in behaviour as someone with variance of 0.1. Variance of ROG and maximum distance from home are not bound between absolute 0 and 1 and can take any positive values, therefore we cannot determine whether increased variance is due to differences in different distances travelled or differences in habituality making the measure unsuitable to compare between individuals and is therefore not presented here.

### 3.3 Interpersonal variability in mobility measures by sex, season and city

Fig. 3 presents the variance in intrapersonal day-to-day correlation coefficients by sex, season and city (weekday data presented in Supplementary Fig. 1). There was some variation in the four mobility measures, however, examination of the interquartile ranges does not indicate substantial differences between sex, season and city.

For example, there was a 17% difference in the variance figures between men and women; but during weekdays males showed 22% more variation in METS correlation coefficients (0.025) than females (0.020) (Full table: Supplementary Table 2 and 3).

By season, the variance in METS remained low but there were some differences in this, with a 40% higher median variability in activity during the Spring (0.035) compared to Autumn (0.025), and 45% greater variability in hourly displacement overall during the Winter season (0.087) compared to Autumn (0.006).

Similarly to sex and season, there was a low variance in METS between cities; a 6% difference in day-to-day variability of activity between Barcelona (0.216) and London (0.204). When examining weekdays only, there was a higher variation between proportion of time at home (81%) and METS (36%), suggesting increased variability in day-to-day mobility patterns during weekdays between cities.

### 3.4 Intrapersonal day-to-day variability in mobility and perceived stress (PSS-4)

The results show there was no association between day-to-day variability in mobility, using six mobility metrics, and stress scores (Table 4). These results were similar when assessing variability for all days and Monday to Friday only.

**Table 4:** Day-to-day variability in mobility behaviours (as measured using 6 mobility metrics) and mental health (PSS-4) (n = 94).

PSS-4 and mobility measures	ROC*		Hourly displacement^		Proportion of time at home^		Time outside the home neighbourhood^		Maximum distance from home*		Energy Expenditure^	
	x2	p	F	p	F	p	F	p	x2	p	F	p
Overall (7 days)	1.08	0.58	2.35	0.10	0.97	0.38	1.08	0.34	1.19	0.55	1.19	0.30
Weekday (Monday to Friday)	0.29	0.86	1.89	0.15	0.48	0.62	1.01	0.37	0.56	0.76	1.97	0.14

*Kruskal-Wallis chi-squared; ^ generalised Levene's.

## 4 Discussion

### 4.1 Key findings

The first two aims of this study were to calculate describe individuals’ patterns of mobility using detailed GPS data, collected from Antwerp, Barcelona and London. The third aim was to explore interpersonal variability in aggregated mobility metrics by sex, season and city. The final aim was to describe day-to-day intrapersonal variability in mobility and their association with perceived stress.

The median daily distance travelled was 12392.5 m (m), and the median METS expended was 1.57. There was significant variation in daily distance travelled, ROG, maximum distance from home and METS between individuals by sex, season and, for proportion of time at home also, by city. Males travelled further distances daily, had a larger maximum distance from home and greater energy expenditure than females, supporting previous research highlighting gender differences in urban mobility (Shaw et al., 2020). Daily energy expenditure was greatest during the Spring period compared to Winter/Autumn; which was expected as prevalence of active transport is lower during this period, which contributes to daily METs (Yang et al., 2011). Individuals living in London travelled further distances daily than those living in Antwerp and Barcelona, which may be due to the variation in city size and links to other research that suggests urban form is associated with daily mobility patterns (Kang et al., 2012).

Within individuals, we found day-to-day hourly displacement, proportion of time at home, time outside the home neighbourhood and METS to be moderately to highly correlated between consecutive days, particularly on weekdays. This suggests that individuals’ day-to-day mobility tends to be similar. That is useful information for those estimating environmental exposures, because it suggests that individuals are creatures of habit. The growth in simulation studies, for example, offers the potential for exploring both our understanding of, and potential interventions in, complex dynamic systems in public health. Approaches such as Agent Based Modelling (ABMs) require behavioural rules for their subjects and a sense of habituality. Mobility data have been used to validate ABMs testing congestion easing policies on public transport in North America (He et al., 2021).

Finally, we did not observe any association between stress and mobility measured using six commonly applied metrics.

### 4.2 Comparison with other literature

We found that participants’ mobility was highly correlated from day to day, particularly during the working week, and this largely echoes the modest existing literature. Research in London found that public transport use for individuals was similarly patterned in the short term (Zhao et al., 2018). The authors used smartcard information from 3210 users with at least 50 weeks of data. They were also able to detect changes in individual travel patterns and found these were largely attributed to life events, such as moving house. An Australian study, also focused on public transport, found a correlation between spatial and temporal similarity matrices calculated using Pearson coefficient (Faroqi et al., 2017), a similar method to ours. They observed that passengers with comparable trip lengths were most likely to have similarity in travel patterns. Similarly, observations of North American vehicle trip patterns found that intrapersonal variability in daily activity sequences was narrow compared to interpersonal variability (Shou & Di, 2018).

Other measures that cannot be computed using mobility data may be more useful to highlight day-to-day variability in behaviours. For example, day-to-day variation for a number of social interaction measures and a greater number of *quality* daily social contacts have been associated with lower subjective stress (Stoffel et al., 2021). Suggesting further research and studies designs are required that combine mobility metrics and social interaction measures.

We did observe some differences between cities, albeit in a sample that was not designed to provide city-to-city comparisons. Travel behaviours have been shown to be influenced by built environment characteristics and urban design, decreased for example by compact, public transport-oriented and pedestrian-friendly environments (Liu et al., 2021). Exploring the relationship between urban form, built environment characteristics and a range of mobility indicators is important. Although we included three major metropolitan cities in our sample, they do have different sizes, urban designs and climates. Size, in particular, might to affect distances travelled from home. Recent GPS studies have shown that the built environment can influence physical activity positively and negatively (Boakye et al., 2021) and there is socioeconomic variation in access to green spaces around the home that are associated with its use (Olsen, Caryl, et al., 2022). Future research should also considering apply weighting to mobility measures as distances travelled are influence by age (Mizen et al., 2020) and travel mode (Hosford et al., 2022).

Similar to studies of heterogeneous populations, we found no association between mobility and stress (Müller et al., 2021), although our ability to assess this was constrained by a small sample size. Müller et al. (2021) found that mobility patterns provide good prediction of mental health outcomes within homogeneous populations whereas the predictions were only marginally more accurate than chance for nationally representative cohorts (Müller et al., 2021). A systematic review found consistent evidence for association between features of mobility such as home stay, location variance and distance moved, and depressive mood symptoms (Rohani et al., 2018). The review by Rohani et al. (2018) highlighted mobility patterns and mental health outcomes as a relatively new field of investigation but one with evidence currently limited by methodological issues in data collation, mood assessment and statistical methods.

We found that our sample followed similar daily patterns of mobility, even in different weeks (seasons) of the study. Specific personality traits might explain more or less habitual behaviours, for example compulsive personalities are associated with an increase in aspects of habitual tendency and those seeking or avoiding situations of novelty or excitement may be more or less likely apply routines to their daily lives (Ersche et al., 2017, 2019). An individual's mobility and their mental health is complex and may provide both positive and negative feedback loops, having depressive symptoms can affect the ability to travel and/or travel could induce stress that aggravates mental health (Mackett, 2021). Travel requires a number of skills that having a mental health condition can affect, such as confidence to take decisions, social interaction, concentration, and information processing, that may subsequently affect the ability to travel (Mackett, 2017). The direction of causation remains uncertain whether active travel makes people happy or happier people are more likely to actively travel (Kroesen & De Vos, 2020). Real-time mobility data from wearable or smart phone devices offer great potential for predicting mental health outcomes or changes in these, future research should ensure a large and representative sample to overcome many of these methodological shortcomings, including their effectiveness for these populations for modelling mental health outcomes.

### 4.3 Strengths and limitations

Our study had several strengths. It provided an international perspective on intra- and inter-personal variation in mobility stemmed from three cities with varying urban design policies and transport cultures. The use of GPS meant robust and consistent measurements of mobility over longer time periods than might be achieved by travel diaries. We were also able to draw on different mobility metrics and explore differences by sex and season. We applied six metrics commonly used within the literature for describing mobility patterns, our data holding many advantages to those using mobility data from telephone network masts by accurately detecting the home location and allowing linkage to sociodemographic information. This allowed us to calculate the distance between each GPS points and a home location, as well as distances between consecutive GPS points. Many existing studies have been based on one location, and/or been focused on one mode or sector of transportation. Whilst this was a small study, one design applied across all sites, the length of wear-time achieved and the accompanying extensive individual-level data capturing participants’ characteristics (such as sex, age, and income) were all assets which enabled novel analyses and insights.

However, our study also had several limitations. We did not formally include measures of the urban form and design, although this may influence travel behaviours it is possibly less likely to impact the variability in daily patterns of behaviour. Future studies should explore various urban designs and how it may influence behavioural patterns. Our study was not designed to provide city-to-city comparisons and had a limited sample in each location, meaning our findings are useful but not definitive given the non-random/representative nature of our sample. Our sample was biased towards both a high income and educated population, both of these factors have been found to affect travel behaviours (Chudyk et al., 2015) and future research should include a more representative sample to explore inequalities in urban mobility. The small and skewed sample did not allow us to explore socioeconomic inequalities in our main outcomes or to fully explore the relationship with mental health outcomes. Due to the invasive nature of inviting participants to complete multiple questionnaires and wear GPS devises, studies of this design often struggle to recruit representative samples. Future studies could explore extracting mobility data from devices in a non-invasive nature, such as mobile phone traces (Calabrese et al., 2013), to improve sample representation biases.

## 5 Conclusions

Using international, objectively measured data we were able to describe and explore individuals’ patterns of mobility, assessing between-group differences and intrapersonal day-to-day variance in six mobility metrics. We found that intrapersonal mobility was highly similar day-to-day, although there was variation between individuals by sex, season and city. Our results suggest that individuals are habitual in their daily mobility patterns and provides important data for those estimating environmental exposures. We did not observe any association between stress and mobility, measured using six commonly applied mobility metrics.

## Funding statement

JO, RM, NN and FC are employed by the MRC/CSO Social and Public Health Sciences Unit, University of Glasgow, and supported by the 10.13039/501100000265Medical Research Council [grant number MC_UU_00022/4] and 10.13039/501100000589Chief Scientist Office [grant number SPHSU19]. FC is supported by an MRC Skills Development Fellowship [MR/T027789/1]. The authors declare that there are no conflicts of interest. ED was supported by a postdoctoral scholarship from 10.13039/501100003130FWO – Research Foundation Flanders. ML held a joint PASTA/VITO PhD scholarship.

This work was supported by the European project Physical Activity through Sustainable Transportation Approaches (PASTA). PASTA (http://www.pastaproject.eu/) was a four-year project funded by the 10.13039/100011102European Union's Seventh Framework Program (EU FP7) under European Commission - Grant Agreement No. 602624.

## Ethics approval

Ethics approval was obtained for all aspects of the study by the local ethics committees in the countries where the work was conducted, and sent to the European Commission before the start of the survey/study.

The following committees approved the study:

• Ethics board of the University Hospital of Antwerp (Belgium) on October 20, 2014
• Clinical Research Ethics Committee of the Municipal Health Care (Barcelona – Spain) on October 1, 2014
• Imperial College Research Ethics Committee (London – UK) on November 20, 2014
• Regional ethical board, situated at the University of Lund (Oerebro – Sweden) on April 9, 2015
• RSM - Roma Servizi per la Mobilità and the Air quality Commission of Roma Capitale Administration (Rome – Italy) on November 24, 2014
• The Austrian Data Processing Register (Vienna – Austria) on September 26, 2014
• Kantonale Ethikkommission Zürich (Switzerland) on October 28, 2014

## CRediT author statement

**Jonathan Olsen:** Conceptualization, Methodology, Formal analysis, Writing - Original Draft. **Natalie Nicholls:** Methodology, Formal analysis, Writing - Review & Editing. **Fiona Caryl:** Methodology, Writing - Review & Editing. **Juan Orjuela Mendoza**: Data Curation, Writing - Review & Editing. **Luc Int Panis:** Data Curation, Writing - Review & Editing, Funding acquisition. **Evi Dons:** Data Curation, Writing - Review & Editing, Funding acquisition. **Michelle Laeremans:** Data Curation, Writing - Review & Editing. **Arnout Standaert:** Data Curation, Writing - Review & Editing, Funding acquisition. **Duncan Lee:** Methodology, Writing - Review & Editing. **Ione Avila-Palencia:** Data Curation, Writing - Review & Editing. **Audrey de Nazelle:** Methodology, Data Curation, Writing - Review & Editing, Funding acquisition. **Mark Nieuwenhuijsen:** Data Curation, Writing - Review & Editing, Funding acquisition. **Richard Mitchell:** Conceptualization, Methodology, Writing - Review & Editing.

## Declaration of competing interest

None.
