# Supplementary material for: Day-to-day intrapersonal variability in mobility patterns and association with perceived stress: A cross-sectional study using GPS from 122 individuals in three European cities
Source: SSM Popul Health. 2022 Jul 16;19:101172. doi: 10.1016/j.ssmph.2022.101172 (PMC9294330; doi:10.1016/j.ssmph.2022.101172)

**Day-to-day intrapersonal variability in mobility patterns and association with perceived stress: a cross-sectional study using GPS from 122 individuals in three European cities.**

**Appendicies**

**List of Appendices**

**Supplementary Tables**

Supplementary Table 1: City characteristics

Supplementary Table 2: Day-to-day variance across all individuals in daily correlation coefficient by season, sex and city: displacement entropy, spatial trajectory from home (m), proportion of time at home, time outside the home neighbourhood and energy expenditure (mets), overall (includes both weekdays and weekends).

Supplementary Table 3: Day-to-day variance across all individuals in daily correlation coefficient by season, sex and city: displacement entropy, spatial trajectory from home (m), proportion of time at home, time outside the home neighbourhood and energy expenditure (mets), weekday only (Weekdays only (excludes Saturday and Sunday).

**Supplementary Figure**

Supplementary Figure 1: Day-to-day variance across all individuals in daily correlation coefficient by season, sex and city: median day-to-day spatial trajectory from home (m) and median day-to-day energy expenditure (mets), weekdays only.

**Supplementary Table 1:** **City characteristics**

| **City** | **Antwerp** | **Barcelona** | **London** |
| --- | --- | --- | --- |
| Size (km2) ^1^ | 204 | 99 | 1594 |
| Population (2018) ^1^ | 523591 | 3665687 | 8866541 |
| Population density (per km2) ^1^ | 2567 | 37027 | 5562 |
| Crude death rate (per 1000 inhabitants) (2016) ^1^ | 9 | 9 | 6 |
| Infant mortality rate (per 1000 live births) (2016) ^1^ | 4 | 2 | 3 |
| Age dependency ratio (population aged 0-19 and 65 and more to population aged 20-64) (2015) ^1^ | 69.4 | 62.1 | 56.7 |
| Share of journeys to work by public transport (%) (2011) ^1^ | 20.7 | 24.3 | 52.6 |
| Share of land (%): Continuous urban fabric^2^* | 10.3 | 27.9 | 2.4 |
| Share of land (%): Industrial, commercial, public, military^2^ | 7.9 | 18.8 | 10.5 |
| Share of land (%): Green urban areas^2^ | 4.6 | 6.0 | 10.1 |
| Share of land (%): Railways, Fast transit roads and associated land^2^ | 7.8 | 2.2 | 1.3 |

***Source:*** *^1^European Commission (2020); ^2^Copernicus (2018)*

*Note: * Continuous urban fabric: > 80% of the land surface is covered by impermeable features like buildings, roads and artificially surfaced areas*

***Supplementary Table 2: Day-to-day variance across all individuals in daily correlation coefficient*** ***by season, sex and city: hourly displacement, spatial trajectory from home (m), proportion of time at home, time outside the home neighbourhood and energy expenditure (mets), overall (includes both weekdays and weekends).***

| **Measure** | **Hourly Displacement (m)** | | **Proportion of time at home (%)** | | **Time outside the home neighbourhood (%)** | | **Energy Expenditure (METs)** | |
| --- | --- | --- | --- | --- | --- | --- | --- | --- |
|  | Median | Range | Median | Range | Median | Range | Median | Range |
| **City** | | | | | | | | |
| Antwerp (n=41; obs=732) | 0.077 | 0.03 to 0.142 | 0.057 | 0.0 to 0.146 | 0.056 | 0.001 to 0.163 | 0.06 | 0.01 to 0.14 |
| Barcelona (n=41; obs=764) | 0.078 | 0.04 to 0.24 | 0.05 | 0.0 to 0.14 | 0.05 | 0.0 to 0.143 | 0.06 | 0.01 to 0.17 |
| London (n=40; obs 783) | 0.073 | 0.05 to 0.26 | 0.041 | 0.0 to 0.13 | 0.045 | 0.0 to 0.186 | 0.10 | 0.01 to 0.28 |
| **Sex** | | | | | | | | |
| Male (n=67; obs=985) | 0.079 | 0.03 to 0.18 | 0.05 | 0.0 to 0.14 | 0.056 | 0.001 to 0.163 | 0.08 | 0.01 to 0.28 |
| Female (n= 55; obs=1294) | 0.073 | 0.04 to 0.26 | 0.05 | 0.0 to 0.146 | 0.047 | 0.0 to 0.186 | 0.06 | 0.01 to 0.18 |
| **Season** | | | | | | | | |
| Winter (obs=724) | 0.087 | 0.01 to 0.26 | 0.033 | 0.0 to 0.176 | 0.038 | 0.0 to 0.187 | 0.0298 | 0.00 to 0.55 |
| Spring (obs=466) | 0.062 | 0.01 to 0.24 | 0.034 | 0.0 to 0.168 | 0.045 | 0.0 to 0.182 | 0.0345 | 0.00 to 0.36 |
| Summer (obs=593) | 0.07 | 0.01 to 0.16 | 0.026 | 0.0 to 0.15 | 0.041 | 0.0 to 0.185 | 0.0266 | 0.00 to 0.39 |
| Autumn (obs=496) | 0.06 | 0.01 to 0.26 | 0.035 | 0.0 to 0.13 | 0.033 | 0.0 to 0.166 | 0.0247 | 0.00 to 0.34 |

**Note: 0 variance indicates no variability identified across individuals’ daily correlation coefficients, the closer to zero, the more regular the daily mobility. Variances are absolute to compare, a person with a variance of 0.2 has twice the amount of variability in behaviour as someone with variance of 0.1.**

***Supplementary Table 3: Day-to-day variance across all individuals in daily correlation coefficient by season, sex and city: hourly displacement, spatial trajectory from home (m), proportion of time at home, time outside the home neighbourhood and energy expenditure (mets), weekday only (Weekdays only (excludes Saturday and Sunday).***

| **Measure** | **Hourly displacement (m)** | | **Proportion of time at home (%)** | | **Time outside the home neighbourhood (%)** | | **Energy Expenditure (METs)** | |
| --- | --- | --- | --- | --- | --- | --- | --- | --- |
|  | Median | Range | Median | Range | Median | Range | Median | Range |
| **City** | | | | | | | | |
| Antwerp (n=41; obs=732) | 0.07 | 0.02 to 0.17 | 0.041 | 0.0 to 0.16 | 0.048 | 0.0 to 0.152 | 0.04 | 0.01 to 0.13 |
| Barcelona (n=41; obs=764) | 0.07 | 0.03 to 0.21 | 0.049 | 0.0 to 0.19 | 0.043 | 0.0 to 0.135 | 0.04 | 0.00 to 0.13 |
| London (n=40; obs 783) | 0.06 | 0.04 to 0.17 | 0.027 | 0.0 to 0.11 | 0.039 | 0.0 to 0.205 | 0.06 | 0.01 to 0.39 |
| **Sex** | | | | | | | | |
| Male (n=67; obs=985) | 0.07 | 0.02 to 0.21 | 0.037 | 0.0 to 0.146 | 0.045 | 0.0 to 0.152 | 0.06 | 0.01 to 0.39 |
| Female (n= 55; obs=1294) | 0.06 | 0.03 to 0.18 | 0.041 | 0.0 to 0.192 | 0.038 | 0.0 to 0.205 | 0.05 | 0.00 to 0.15 |
| **Season** | | | | | | | | |
| Winter (obs=724) | 0.05 | 0.01 to 0.57 | 0.019 | 0.0 to 0.206 | 0.023 | 0.0 to 0.183 | 0.034 | 0.00 to 0.69 |
| Spring (obs=466) | 0.04 | 0.01 to 0.31 | 0.027 | 0.0 to 0.248 | 0.033 | 0.0 to 0.216 | 0.037 | 0.00 to 0.70 |
| Summer (obs=593) | 0.05 | 0.01 to 0.16 | 0.012 | 0.0 to 0.177 | 0.028 | 0.0 to 0.18 | 0.024 | 0.00 to 0.34 |
| Autumn (obs=496) | 0.06 | 0.01 to 0.35 | 0.023 | 0.0 to 0.14 | 0.025 | 0.0 to 0.172 | 0.025 | 0.00 to 0.15 |

**Note: 0 variance indicates no variability identified across individuals’ daily correlation coefficients, the closer to zero, the more regular the daily mobility. Variances are absolute to compare, a person with a variance of 0.2 has twice the amount of variability in behaviour as someone with variance of 0.1.**

***Supplementary Figure 1: Day-to-day variance across all individuals in daily correlation coefficient by season, sex and city:*** ***hourly displacement, spatial trajectory from home (m), proportion of time at home, time outside the home neighbourhood and energy expenditure (mets), weekdays only.***


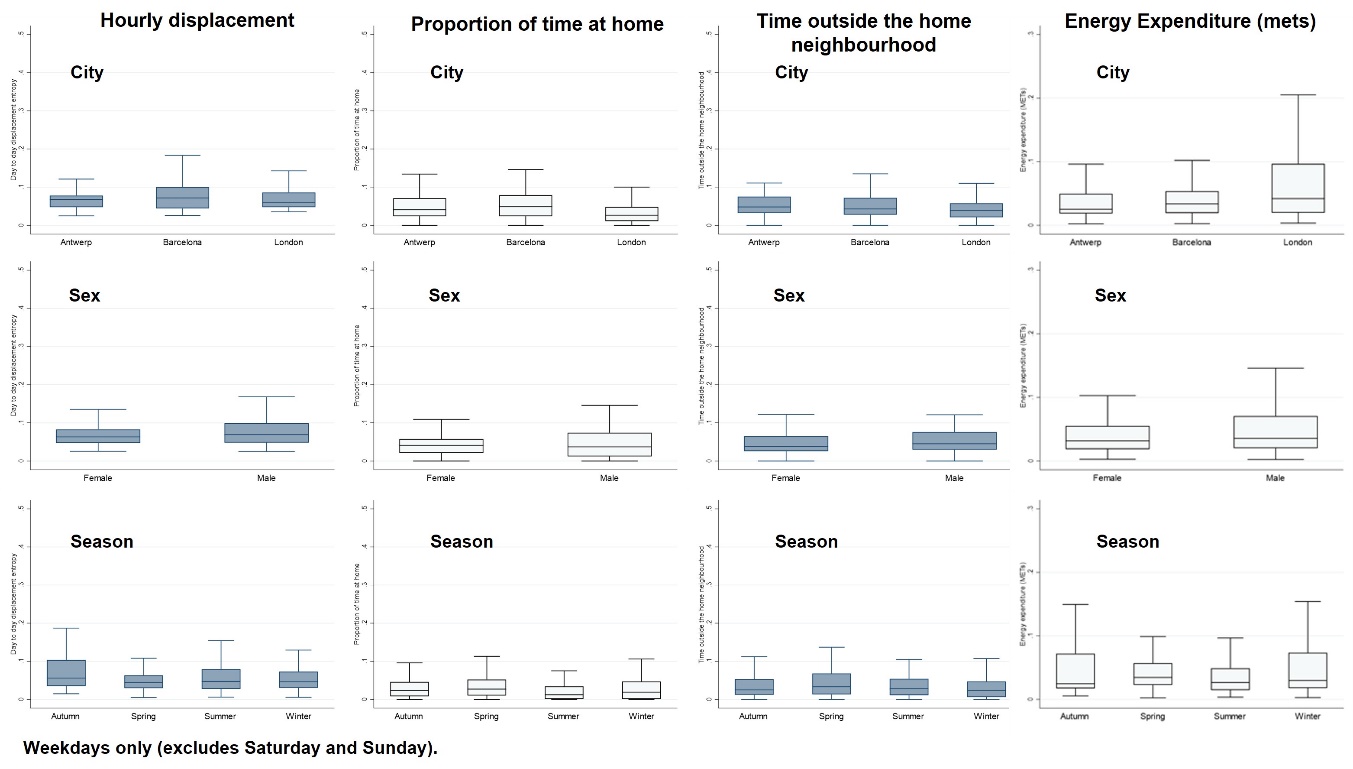

Supplement: Multimedia component 1 [file mmc1.docx]
